# Association of serum cystatin C levels with mortality in patients with acute type A aortic dissection

**DOI:** 10.18632/oncotarget.20593

**Published:** 2017-08-30

**Authors:** Wei-Zhong Feng, Jun-Qing Zhou, Guang-Mao Yu, Yong Zeng, Peng Xu

**Affiliations:** ^1^ Department of Cardiothoracic Surgery, Shaoxing People's Hospital, Shaoxing Hospital of Zhejiang University, Shaoxing 312000, Zhejiang Province, China

**Keywords:** acute type A aortic dissection, cystatin C, high sensitive C-reactive protein, mortality, prognosis

## Abstract

Increased serum cystatin C levels are related to the prognosis of cardiovascular diseases. This study aims to investigate the effect of admission serum cystatin C levels on short- and long-term mortality in patients with acute type A aortic dissection (ATAAD). From 2010 to 2014, 136 consecutive patients with ATAAD were enrolled and followed up. Clinical data and laboratory assays including were measured. During a median follow-up of 198.7 days, the short-term mortality (30-days) was 20.6%, whereas the long-term death rate was 10.2%. We identified that the expression of cystatin C and high-sensitivity C-reactive protein (hs-CRP) in the dying patients was higher than in the surviving patients (*P* < 0.01). Hs-CRP (HR = 1.41, 95% CI: 1.03–2.59, *P* = 0.037) was an independent risk factor of short-term death determined by univariate and multivariate Cox analyses. No impact of cystatin C was observed on the short-term mortality. For long-term mortality, cystatin C (HR = 1.49, 95% CI: 1.10–7.36, *P* = 0.013) was identified as an independent predictor at above the cut-off value ≥ 1.10 mg/L. ROC analysis showed the AUC values of cystatin C and hs-CRP were 0.772 (95% CI, 0.692–0.839) and 0.640 (95% CI, 0.574–0.739), respectively, in the prediction of long-term death. The combined AUC value of cystatin C and hs-CRP was 0.883 (95% CI, 0.826–0.935; *P* < 0.01). Taken together, high cystatin C levels (≥ 1.10 mg/L) on admission are independently associated with the long-term mortality in patients with ATAAD.

## INTRODUCTION

Acute aortic dissection (AAD) is a devastating cardiovascular condition with an incidence of about 2.6–3.6 cases per 100,000 people per year [1]. AAD is characterized by rapid development of an intimal flap, which is caused by blood flow into the media that divides the intima and the adventitia. This intimal flap separates the true lumen from a false lumen [2]. Acute type-A aortic dissection (ATAAD), when the ascending aortic thoracic tract is involved, occurs in almost 75% of patients with AAD. This can lead to a mortality rate reaching 90% if untreated and usually requires swift open surgical repair [3]. Thus, identification of the predictors for short- or long-term mortality of patients with ATAAD may contribute to reduced mortality. Concentrations of white blood cell count (WBCc) [4], C-reactive protein (CRP) [5], D-dimer [6], cardiac troponin T [7] and matrix metalloproteinases [8] have shown to be associated with the prognosis of AAD.

Cystatin C is a type of cysteine protease inhibitor whose levels increase much earlier than those of urea and creatinine when renal function declines [9, 10]. Previous studies have also illustrated an important role of cystatin C in arterial remodeling and atherogenesis [11]. More importantly, many studies have shown that an elevated serum cystatin C level was independently associated with an increased risk of cardiovascular or all-cause mortality [12, 13], and this predictive function may not only result from a marker for detection of renal impairment [14]. To date, no study has determined the prognostic significance of cystatin C in patients with ATAAD. Therefore, this study was designed to determine the diagnostic value of cystatin C in predicting the short- and long-term prognosis of patients with ATAAD.

## RESULTS

### Baseline patient demographics

A total of 136 patients were enrolled in this study, including 77 males and 59 females, with an average age of 53.7 ± 10.3 years. Patient demographics and baseline characteristics are shown in Table 1. After the follow-up period, 97 patients survived and 39 died (28 patients died during short-term follow up and 11 during long-term). The causes of death including aortic rupture (20), myocardial infarction (4), heart failure (6), stroke (4) and valvular dysfunction (5). In our findings, the ascending aorta diameter was larger in the death group when compared to the survival group (*P* = 0.034). Patients who received surgical treatment were more likely to survive (*P* < 0.001). No significant differences in patients’ age, sex, body mass index, and cardiovascular risks such as hypertension and diabetes mellitus were observed between the death and survival patient groups.

**Table 1:** Baseline characteristics of patients with type A acute aortic dissection (AAD)

Clinical variables	Survival (*n* = 97)	Death(*n* = 39)	Statistics	*P*-value
Age(years)	52.8 ± 9.6	54.6 ± 11.5	0.933^a^	0.352
Male Sex *n* (%)	57(58.8%)	20(51.3%)	0.192^b^	0.660
Body mass index (kg/m2)	24.7 ± 3.6	25.4 ± 2.8	1.088^a^	0.278
History of smoking	31(31.9%)	18(46.2%)	2.678^b^	0.101
Hypertension	65(67.0%)	31(79.5%)	1.528^b^	0.216
Diabetes mellitus	19(19.6%)	12(30.8%)	1.392^b^	0.238
Admission SBP (mmHg)	164.5 ± 25.3	170.2 ± 26.8	1.168^a^	0.242
Admission SBP (mmHg)	79.2 ± 14.6	82.8 ± 19.2	1.184^a^	0.238
AAD, mm	44.6 ± 6.1	47.0 ± 5.5	2.132^a^	0.034
Aspirin	16(16.5%)	6(15.4%)	0.009^b^	0.922
Nitroglycerin	38(39.2%)	11(28.2%)	1.015^b^	0.314
Surgical treatment, *n* (%)	79(81.4%)	20 (51.3%)	11.30^b^	< 0.001
Total cholesterol (mmol/L)	165.3 ± 24.8	170.0 ± 30.3	0.936^a^	0.351
LDL-C (mg/dL )	95.3 ± 18.7	97.2 ± 23.1	0.499^a^	0.618
WBCc (×10^9^ cells/L)	12.8 ± 4.0	14.7 ± 4.8	2.362^a^	0.019
hs-CRP (mg/L)	4.2 ± 2.3	5.8 ± 2.1	3.285^a^	< 0.001
D-dimer (μg/mL)	5.9 ± 3.0	7.6 ± 3.5	2.864^a^	0.005
hs-cTnI (ng/mL)	0.011 ± 0.010	0.029 ± 0.021	6.769^a^	< 0.001
BNP (pg/ml)	430 ± 402	584 ± 415	2.002^a^	0.047
Serum Creatinine (μmol/L)	93.1 ± 14.5	95.5 ± 18.9	0.797^a^	0.426
cystatin C (mg/L)	0.89 ± 0.3	1.20 ± 0.5	4.444^a^	< 0.001

Note: Data are expressed as mean ± SD. a = *t* value; b = χ^2^ value; SBP: systolic blood pressure; DBP: diastolic blood pressure; AAD: ascending aorta diameter; LDL-C: low density lipoprotein- cholesterol; WBCc: white blood cell count; hs-cTnI: high-sensitivity troponin I; BNP: Brain natriuretic peptide; hs-CRP: high sensitive C-reactive protein.

**Figure 1:**
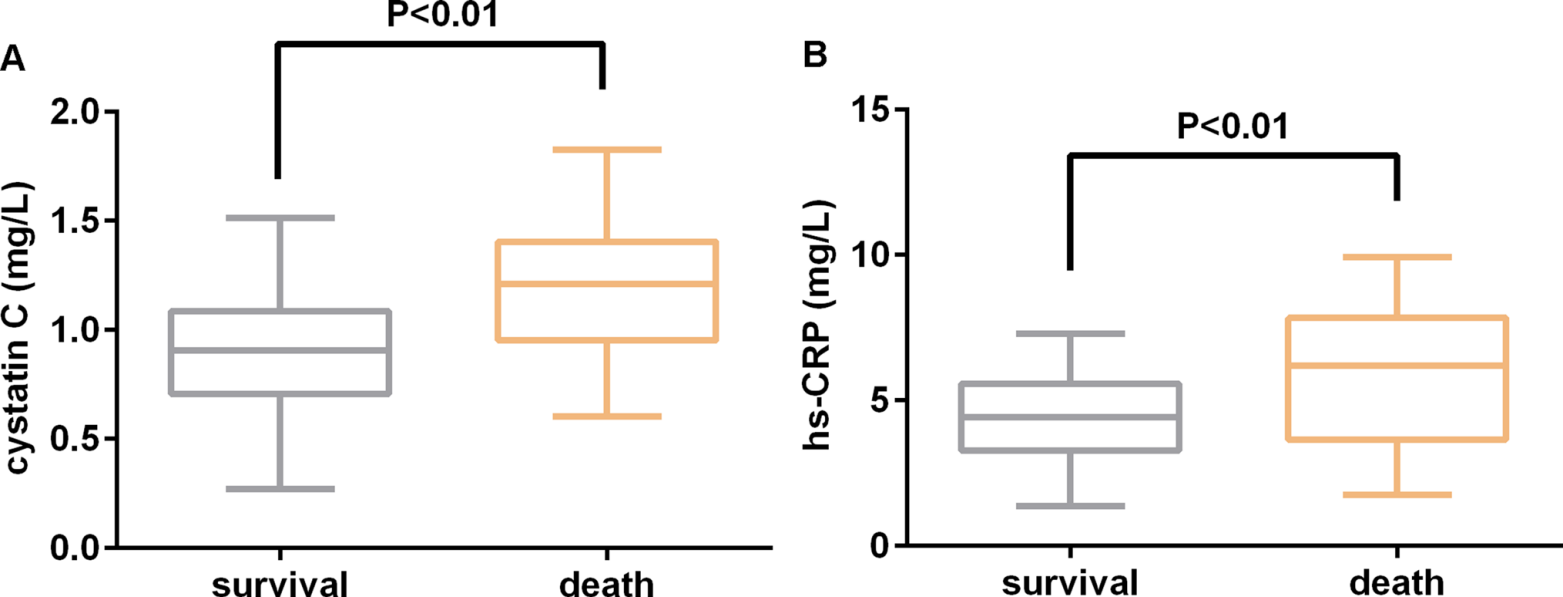
Comparison of (**A**) serum cystatin C and high-sensitivity C-reactive protein (hs-CRP) (**B**) values between survival and death patients admitted with acute Type A aortic dissection.

With respect to laboratory findings, WBCc (14.7 ± 4.8 × 10^9^ vs 12.8 ± 4.0 × 10^9^ cells/L, *P* = 0.019), hs-CRP (5.8 ± 2.1 vs 4.2 ± 2.3 mg/L, *P* < 0.001), D-dimer (7.6 ± 3.5 vs 5.9 ± 3.0 μg/mL, *P* = 0.005), high-sensitivity troponin I (hs-cTnI) (0.029 ± 0.021 vs 0.011 ± 0.010 ng/mL, *P* < 0.001), BNP (584 ± 415 vs 430 ± 402 pg/ml, *P* = 0.047), and cystatin C (1.20 ± 0.5 vs 0.89 ± 0.3 mg/L, *P* < 0.001) concentrations were significantly higher in the death group than in the survival group. The comparison of cystatin C and hs-CRP levels between the two groups are shown in Figure 1.

### Correlation of cystatin C with hs-CRP in ATAAD

**Figure 2:**
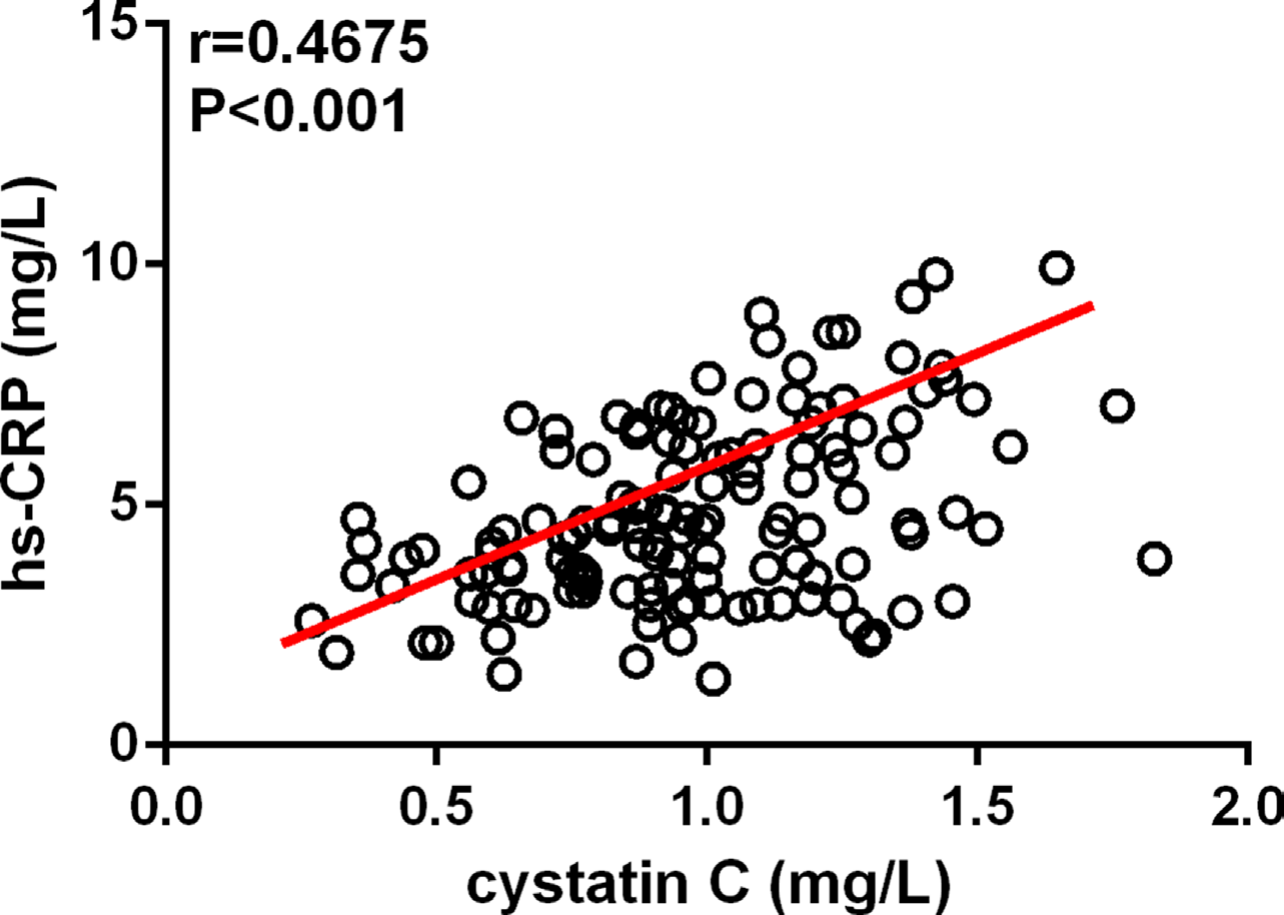
Cystatin C levels showed a positive correlation with high-sensitivity C-reactive protein (hs-CRP) levels in patients with acute type A aortic dissection detected by the Spearman correlation

As shown in Figure 2, serum cystatin C levels existed positive correlation to hs-CRP concentrations, *r* = 0.4675 (*P* < 0.001), which were analyzed by the spearman correlation; serum cystatin C was also correlated with WBCc (*r* = 0.2305, *P* = 0.001), but not D-dimer (*r* = 0.1067, *P* = 0.134; not shown).

### Analysis of risk factors for short- and long-term mortality

**Figure 3:**
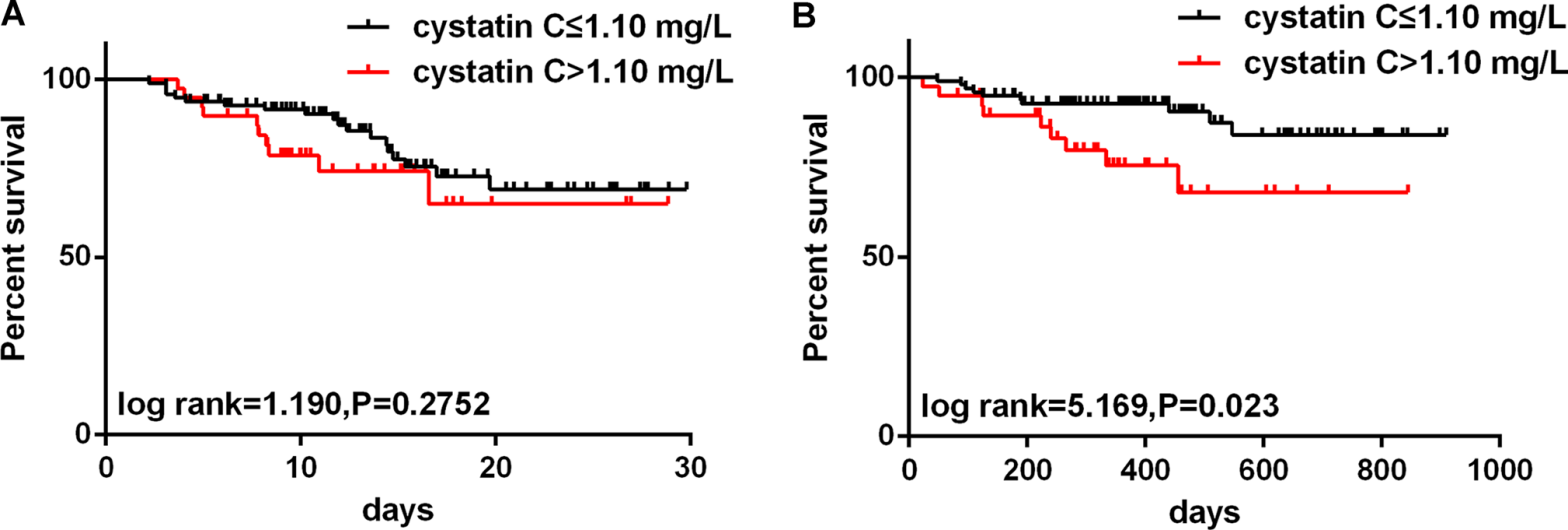
Kaplan-Meier analyses for short- and long-term mortality according to admission cystatin C (cut-off: 1.10 mg/L) values (**A**) Short-term survival curves according to the cystatin C cut-off values (1.10 mg/L). (**B**) Long-term survival curves according to the cystatin C cut-off values (1.10 mg/L).

As shown in Figure 3A, Kaplan-Meier survival curves stratified by admission serum cystatin C levels showed no difference in short-term survival detected by the log rank test (*P* = 0.3917). Interestingly, the log rank test identified a significant difference in long-term survival among the patients stratified by serum cystatin C (Figure 3B, *P* = 0.006).

Univariate and multivariate Cox analyses for short- and long-term mortality in the enrolled patients are presented in Table 2 and Table 3, respectively. The data revealed that seven variables were associated with short-term mortality (20.6%, 28/136) including ascending aorta diameter, surgical treatment, WBCc, hs-CRP, D-dimer, hs-cTnI, and cystatin C. After multivariable adjustment, hs-CRP (HR = 1.41, 95% CI: 1.03–2.59, *P* = 0.037) was an independent risk factor, whereas surgical treatment (HR = 0.12, 95% CI:0.01–0.41, *P* < 0.001) was an independent protective factor for short-term mortality (Table 2). During the long-term follow-up, the all-cause mortality was 10.2% (11/108). Univariate analysis revealed three associated variables and multivariable analysis indicated that serum cystatin C (HR = 1.49, 95% CI: 1.10–7.36, *P* = 0.013) and surgical treatment (HR=0.22, 95% CI:0.03–0.57, *P* < 0.001) were independent predictors of long-term mortality (Table 3).

**Table 2:** Effects of various variables on short-term mortality in univariate and multivariate logistic regression analyses

Variable	Univariable		Multivariable	
	OR (95% CI)	*P*-value	OR (95% CI)	*P*-value
Age > 65 years	1.21 (0.82–1.89)	0.312		
History of smoking	1.66 (0.65–4.67)	0.553		
Admission SBP (> 140 mmHg)	1.31 (0.95–2.09)	0.350		
Admission DBP (> 90 mmHg)	0.86 (0.31–1.58)	0.441		
AAD, mm	1.34 (0.99–3.31)	0.042	1.22 (0.97–1.86)	0.134
Surgical treatment	0.07 (0.01–0.35)	< 0.001	0.12 (0.01–0.41)	< 0.001
WBCc (×10^9^ cells/L)	1.98 (0.99–4.58)	0.004	1.19 (0.91–3.16)	0.073
hs-CRP (mg/L)	2.11 (1.72–8.35)	0.012	1.41 (1.03–2.59)	0.037
D-dimer (μg/mL)	4.28 (2.10–12.92)	< 0.001	3.35 (0.82–9.41)	0.109
hs-cTnI (ng/mL)	1.65 (1.05-1.50)	0.017	1.20 (0.96–1.33)	0.215
BNP (pg/ml)	1.04 (0.85-2.90)	0.185		
Serum Creatinine (μmol/L)	1.79 (0.77–4.46)	0.259		
cystatin C (mg/L)	2.54 (1.14–7.86)	0.002	1.67 (0.84–2.87)	0.238

Note: SBP: systolic blood pressure; DBP: diastolic blood pressure; AAD: acute aortic dissection; WBCc: white blood cell count; hs-cTnI: high-sensitivity troponin I; BNP: Brain natriuretic peptide; hs-CRP: high sensitive C-reactive protein. OR: Odds ratio; CI: Confidence interval.

**Table 3:** Effects of various variables on long-term mortality in univariate and multivariate logistic regression analyses

Variable	Univariable		Multivariable	
	OR (95% CI)	P-value	OR (95% CI)	P-value
Age > 65 years	1.51 (0.95–3.88)	0.032	1.20 (0.83–2.20)	0.406
History of smoking	1.44 (0.71–4.59)	0.318		
Admission SBP (> 140 mmHg)	0.98 (0.42–3.76)	0.417		
Admission DBP (> 90 mmHg)	0.74 (0.25–2.52)	0.671		
AAD, mm	1.14 (0.93–2.29)	0.172		
Surgical treatment	0.15 (0.03–0.41)	< 0.001	0.22 (0.03–0.57)	< 0.001
WBCc (×10^9^ cells/L)	1.38 (0.74–6.42)	0.211		
hs-CRP (mg/L)	1.21 (0.82–5.49)	0.134		
D-dimer (μg/mL)	1.98 (0.86–4.67)	0.320		
hs-cTnI (ng/mL)	1.10 (0.91–1.21)	0.147		
BNP (pg/ml)	1.02 (0.78–1.06)	0.343		
Serum Creatinine (μmol/L)	0.93 (0.47–2.44)	0.604		
cystatin C (mg/L)	1.94 (1.25–9.03)	< 0.001	1.49 (1.10–7.36)	0.013

Note: SBP: systolic blood pressure; DBP: diastolic blood pressure; AAD: acute aortic dissection; WBCc: white blood cell count; hs-cTnI: high-sensitivity troponin I; BNP: Brain natriuretic peptide; hs-CRP: high sensitive C-reactive protein. OR: Odds ratio; CI: Confidence interval.

### Diagnostic value of cystatin C in predicting mortality

**Figure 4:**
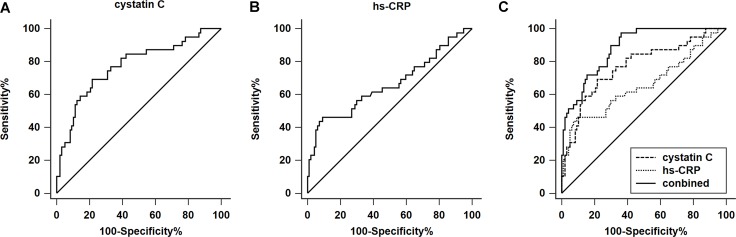
ROC analysis of serum cystatin C and high-sensitivity C-reactive protein (hs-CRP) in the long-term mortality of patients with acute type A aortic dissection (**A**) In the ROC analysis of serum cystatin C, the AUC value is 0.772 (95% CI: 0.692–0.839) with a sensitivity and specificity of 78.53% and 69.23%, respectively. The cut-off value is 1.10 mg/L. (**B**) In the ROC analysis of hs-CRP, the AUC value is 0.640 (95% CI: 0.574–0.739) with a sensitivity and specificity of 86.72% and 46.51%, respectively. The cut-off value is 5.57 mg/L. (**C**) The AUC value of combined (cystatin C+ hs-CRP) is 0.883 (95% CI: 0.826–0.935) with a sensitivity and specificity of 97.44% and 65.92%, respectively. The cut-off value is -1.51. (combined = −8.40 + 0.62 * hs-CRP + 0.45 * cystatin C).

Receiver operating characteristic (ROC) curves were used to further investigate the predictive power of cystatin C in evaluating long-term mortality. The results indicated that the area under the curve (AUC) value of cystatin C was 0.772 (95% CI, 0.692–0.839; Figure 4A), while the AUC of hs-CRP was 0.640 (95% CI, 0.574–0.739; Figure 4B). When cystatin C was ≥ 1.10 mg/L, the sensitivity and specificity in predicting long-term death were 78.53% and 68.23%, respectively. When hs-CRP was ≥ 5.76 mg/L, the sensitivity and specificity were 86.72% and 46.51%, respectively. Furthermore, the ROC curve comparison revealed a higher prognostic value of cystatin C + hs-CRP compared with cystatin C or hs-CRP alone (*P* < 0.01) as shown in Figure 4C. Cystatin C + hs-CRP yielded the highest AUC values at 0.883 (95% CI, 0.826–0.935), with a sensitivity of 97.44% and a specificity of 65.92% (Figure 4C).

## DISCUSSION

This study identified that serum cystatin C levels in patients with ATAAD had a good correlation with hs-CRP levels. Although no association was found between serum cystatin C and short-term mortality, our data showed when using the cut-off value ≥ 1.10 mg/L, cystatin C was an independent risk factor for long-term prognosis. The sensitivity and specificity in predicting long-term mortality were 78.53% and 68.23, respectively, suggesting that cystatin C on admission is an independent predictor of long-term mortality in patients with ATAAD.

AAD is the main cause of cardiovascular morbidity and mortality. Although no epidemiological data have been confirmed, the number of patients with AAD has increased in China in recent years [15]. ATAAD owns high in-hospital mortality due to potentially fatal complications, such as coronary involvement, even in centers that have extensive expertise and interest in the treatment of high-risk patients [16]. The reoccurrence of aortic dissection in the chronic phase may also contribute to the high mortality [17]. Therefore, classifying high-risk patients with a poor long-term prognosis is clinically important.

Cystatin C is a non-glycosylated basic protein produced at a constant rate by investigated nucleated cells. It is considered as a novel marker for renal dysfunction, which is better than serum creatinine, especially for mild renal impairment [18, 19]. Renal impairment is clearly an independent risk factor for cardiovascular disease and a close relationship has been established between cystatin C and various subsets of cardiovascular diseases, including coronary heart disease [20], acute coronary syndrome [21] and heart failure [10]. Pascual et al. suggested that cystatin C independently predicts death or heart failure rehospitalization with greater accuracy than creatinine [22]. Various studies found that cystatin C is also an important prognostic factor of a poor outcome in patients with acute coronary syndrome [23, 24]. These all demonstrated the prognostic significance of cystatin C in cardiovascular disease. In this work, admission serum cystatin C was found to be potentially related to poor long-term outcomes in patients with ATAAD. We showed patients with higher levels of admission cystatin C (≥ 1.10 mg/L) the exerted long survival time than lower ones. Because cystatin C plays a role in the balance between cysteine proteases and their inhibitor that favors remodeling of the vascular wall [11], a potential explanation of the association between the serum cystatin C value and the long-term outcomes in ATAAD is that elevated cystatin C values might affect the remodeling of the dissected aortic wall. Studies by Sukhova et al. [25] and Liu et al. [11] supported our hypothesis. As for short-term mortality, cystatin C showed no sufficient power to predict outcomes when classified using the cut-off value at 1.10 mg/L. A recent study by Mori et al. showed that thoracic endovascular aortic repair of lesions in the descending thoracic aorta has no influence on the level of cystatin C in AAD patients [26]. The expression levels of cystatin C were immune to AAD therapy in a short time, which may explain our findings that cystatin C could be used to predict long-term rather than short-term mortality.

In addition, cystatin C was found to correlate with hs-CRP (markers of inflammation), as well as WBCc. One potential reason for this correlation is that like many other markers of inflammation, its serum concentration may be higher in patients with decreased renal clearance. However, few patients in our cohort experienced severe renal impairment. Another reason may be that cystatin C is a marker more than for renal function and a marker of inflammation [27]. Although numerous studies reported that the cystatin C level is produced at a constant rate and not affected by such factors, we found that in patients with ATAAD, serum cystatin C level had a good correlation with hs-CRP levels. This was supported by the finding by Knight et al. that serum cystatin C appears to be influenced by factors other than renal function alone [28]. Inflammation has been reported to be involved in the pathogenesis of AAD and associated with outcomes [4, 29, 30]. CRP is a well-established inflammatory marker [31]. In line with previous studies, hs-CRP (HR = 1.41, 95% CI: 1.03–2.59) was found to be a predictive marker for short-term morality. Nevertheless, there was no association between long-term all-cause mortality and hs-CRP levels, which may be due to hs-CRP as a marker of the acute phase [30, 32]. Contrarily, Mori et al. found admission CR*P* values might be a useful marker to predict the long-term outcome in acute aortic dissection and they hypothesized that elevated admission CR*P* values might also affect the remodeling of the dissected aortic wall [33]. This discrepancy might because they investigate the prognostic value of CRP in both type A and type B patients. Thus, further studies with more patients from multicenter are needed to determine the exact significance of CRP in acute aortic dissection.

It is likely that no single biomarker will be perfectly predictive, but identifying the optimum combinations to develop risk stratification may determine whether relevant therapies affecting these biomarkers have the potential to reduce mortality. We finally found that the combined use of both cystatin C and hs-CRP levels could increase the sensitivity in assessing long-term mortality up to 97.44%. Therefore, according to our data, it may be reasonable to take both cystatin C and hs-CRP into account as the prognostic markers in patients with ATAAD because of the high sensitivity demonstrated. The data suggesting that if both admission cystatin C and hs-CRP levels were above the cut-off levels in ATAAD patients, the risk of death was significantly increased.

Some limitations of the present study need to be addressed. First, we did not obtain the cystatin C profiles at regular intervals during the follow up because of high test costs. As a prognostic marker, it needs more information about dynamic alteration of cystatin C. Second, no patients with end-stage renal disease were included in our cohort, and other studies are needed to evaluate how informative this biomarker will be in individuals with renal dysfunction, as it always occurs in progression of this disease [34]. Furthermore, the cystatin C levels were included as categorical variables, and there still exerts residual confounding. Thus, larger prospective multicenter studies are needed to further confirm our findings.

In conclusion, our study preliminary demonstrated that serum cystatin C ≥ 1.10 mg/L and hs-CRP ≥ 5.76 mg/L were important risk factors and independently associated with ATAAD long-term mortality. Furthermore, the combination of serum cystatin C and hs-CRP is a potentially useful biomarker with high sensitivity in predicting long-term death in patients with ATAAD in clinical settings.

## MATERIALS AND METHODS

### Study population

We enrolled 136 consecutive patients with ATAAD at Shaoxing Hospital between February 2010 and December 2014. Each patient was diagnosed with ATAAD according to the standard guideline [35] assisting with clinical symptoms, history, ultrasound, computed tomography angiography, ae well as coronary angiography. Acute dissection is defined as within 2 weeks of symptom onset [36]. Exclusion criteria: a) a history of AD episodes, traumatic AD, aortic surgery, and/or endovascular interventions; b) severe organ dysfunction, such as liver or kidney failure; c) Marfan syndrome and those treated with steroids for long-term use; d) incomplete information for an earlier death during the study. The standard data entry form included information on patient demographics, history, complications, imaging study results, details of medical and surgical treatment (including emergency and selective surgery), results of laboratory assays, and clinical outcomes. Patients were followed up every day in-hospital (outcome of death caused by aortic rupture, perioperative death or other causes) and a follow up protocol was conducted after discharge that included clinical assessment and imaging of the aorta (the endpoint was the occurrence of death or loss to follow-up). The short-term mortality was defined as death within 30 days. The mean follow-up time was 198.7 (2–909) days. Hypertension was defined as a systolic blood pressure > 140 mm Hg and/or diastolic blood pressure > 90 mm Hg or the use of antihypertensive drugs. The definition of diabetes mellitus was that HbA1c ≥ 6.5 %, random plasma glucose ≥ 200 mg/dl, fasting plasma glucose ≥ 126 mg/dl or OGTT 2-hour glucose in venous plasma ≥ 200 mg/dl, or was receiving hypoglycemic treatments. The study was approved by the ethics committee of the Shaoxing People's Hospital, and written consent was obtained from each subject.

### Serum collection and testing

Venous blood samples were drawn into ethylenediaminetetra-acetic acid (EDTA)-containing tubes from all patients at the first day upon admission before administration of any medication. Supernatants were obtained within 1 h after centrifugation (4°C, 3500 r/min, 10 min) and the serum and plasma were immediately stored at −80°C until further analysis. The serum levels of cystatin C were measured using enzyme-linked immunosorbent assay kits (Yanjin Biological, Shanghai, People's Republic of China) as previously described [37]. Plasma hs-CRP levels were measured via the colorimetric method with a chemistry analyzer (AU5800 Analyzer, Beckman Coulter, Brea, CA). Total cholesterol, white blood cell count (WBCc), low-density lipoprotein cholesterol (LDL-C), D-dimer, and serum creatinine were determined using standard quantitative assay techniques in the hospital clinical laboratory center according to the manufacturers’ instructions.

### Statistical analysis

Continuous variables were presented as mean ± standard deviation (SD) or median and interquartile range, and categorical variables were displayed as counts or percentages. Student's *t*-test or Mann-Whitney test was used for the analysis of continuous variables and χ^2^-test for categorical variables. The correlation between cystatin C and hs-CRP was determined by the spearman rank correlation. Short- and long-term outcomes were determined using the Kaplan-Meier method and compared using the log-rank test. A logistic regression model was constructed for the multivariable analysis for markers, and cystatin C + hs-CRP (cystatin C + hs-CRP = −8.40 + 0.62* hs-CRP + 0.45* cystatin C) was then generated. Receiver operating characteristic (ROC) analysis was used to compare the prognostic value of cystatin C and D-dimer. SPSS 19.0 (SPSS Inc, Chicago, IL) and GraphPad Prism 6.5 (GraphPad Software Inc., CA) were used to perform statistical analysis. *P* value < 0.05 was considered statistically significant.
